# Assessment of Ocular Surface Damage during the Course of Type 2 Diabetes Mellitus

**DOI:** 10.1155/2018/1206808

**Published:** 2018-07-15

**Authors:** Fanglin He, Zhanlin Zhao, Yan Liu, Linna Lu, Yao Fu

**Affiliations:** ^1^Department of Ophthalmology, Shanghai Ninth People's Hospital, Shanghai Jiaotong University School of Medicine, Shanghai, China; ^2^Shanghai Key Laboratory of Orbital Disease and Ocular Oncology, Shanghai, China

## Abstract

**Purpose:**

To investigate the impact of disease duration on the ocular surface during the course of type 2 diabetes mellitus compared with nondiabetic controls.

**Methods:**

One hundred twenty diabetic patients were divided into three groups according to disease duration: less than 5 years, 5–10 years, and over 10 years. All eyes were imaged using a corneal topographer (Oculus Keratograph 5M). Tear film measurements and meibography were also recorded. Meibomian gland changes were scored from 0 to 6 (meiboscore).

**Results:**

The noninvasive breakup time first (NIKBUT-1st) and noninvasive breakup time average (NIKBUT-avg) were significantly shorter in the over 10 years diabetic group compared with the control group (*P*=0.0056  and  *P*=0.010, resp.). Tear meniscus height (TMH) was significantly lower in the over 10 years diabetic group compared with the control group (*P*=0.0016) and the 5 years group (*P*=0.0061). We also found that more patients in the over 10 years diabetic group showed bulbar and limbal hyperemia compared with the control group (bulbar hyperemia: *P*=0.049; limbal hyperemia: *P*=0.026). The meiboscore in the over 10 years diabetic group was significantly higher compared with the other three groups (*P* < 0.05). Bulbar hyperemia showed a significant negative correlation with NIKBUT-1st in the over 10 years diabetic group (*r*=−0.35  and  *P* < 0.05).

**Conclusion:**

Ocular surface damage in long-term type 2 diabetes is more severe than that in patients with shorter disease duration.

## 1. Introduction

Type 2 diabetes mellitus is one of the largest public health problems worldwide, especially in developing nations such as China [1], due to changes in lifestyle and diet preferences in recent years. Diabetes is often associated with macrovascular abnormalities. Although retinal complications of diabetes are well recognized and can act as a predictive index for the disease course [2], the effects of diabetes on the ocular surface are poorly understood.

However, according to a clinical study, up to 73.6% of type 2 diabetic patients suffer from corneal complications, such as punctate keratopathy, endothelial dystrophy, and recurrent erosions [3]. In particular, diabetic patients often complain of dry eye symptoms, including dryness, burning, redness, pain, ocular irritation, and easily fatigued eyes. The International Dry Eye Workshop 2017 classified diabetes as a risk factor for aqueous-deficient dry eye [4]. During the course of diabetes, microvascular damage to the lacrimal gland due to hyperglycemia, reduced lacrimal innervation as a result of autonomic neuropathy, reduced trophic support to lacrimal tissue, and reduced reflex tearing due to impairment of corneal sensitivity all contribute to the altered tear film status in diabetic patients [4].

In contrast to aqueous-deficient dry eye, which is usually caused by a lack of tear production, evaporative dry eye is due to lid-related and ocular surface-related causes such as meibomian gland dysfunction (MGD) and is more frequent [5]. It is acknowledged that the clinical characteristics of both aqueous-deficient and evaporative dry eye are present in patients as dry eye disease progresses. In addition, a large epidemiologic study in Spain [6] suggested that diabetes was associated with MGD, the major contributor to dry eye according to clinical studies [7, 8].

Corneal changes usually depend on the type, duration, and compensation of diabetes mellitus. In this study, we aimed to analyze the tear film stability and morphological changes in meibomian glands using keratography in diabetic patients compared with nondiabetic controls and to better understand the impact of disease duration on the ocular surface during the course of type 2 diabetes mellitus.

## 2. Materials and Methods

### 2.1. Subjects

The study was approved by the Investigational Review Board of Shanghai Ninth People's Hospital, Shanghai Jiaotong University School of Medicine, Shanghai, China. All subjects enrolled were informed of the aims of this study.

One hundred twenty patients diagnosed with type 2 diabetes mellitus met the inclusion criteria and were enrolled in the study at the Shanghai Ninth People's Hospital between April 2016 and January 2017. Data were obtained from the right eye of each subject unless this eye was excluded, in which case data were collected from the left eye. The inclusion criteria were as follows: at least 40 years of age and willingness to participate in the study. Patients were excluded if they used topical medications; wore contact lenses; had undergone ocular surgery in the past year or had evidence of other ocular surface diseases; had active ocular infection, inflammation, or systemic disease; or were taking medications that would alter the ocular surface. All diabetic patients had a blood glucose level within the normal range. Diabetic patients were divided into the following three groups according to duration of diabetes since the first diagnosis: less than 5 years, 5–10 years, or more than 10 years. Forty subjects were recruited as nondiabetic controls. The exclusion criteria were similar to those of the control group. Fasting blood glucose was measured to rule out diabetes even in those without a history of diabetes. All patients underwent noninvasive ocular surface examinations in the following order: tear meniscus height (TMH), noninvasive tear film breakup time, bulbar and limbal hyperemia, and grading of meibomian gland loss with the Oculus Keratograph 5M (Wetzlar, Germany). All patients were examined by the same physician (He FL).

All diabetic patients were from the Department of Endocrinology in our hospital. These patients had been followed up over a long period, and disease duration was confirmed by their professional endocrinologists. The data in this article are reliable and were obtained from medical files. The groups were matched by age.

### 2.2. Oculus Keratograph 5M

Oculus Keratograph 5M can provide automated measurements of tear film dynamics and meibographic images using infrared light without topical anesthetic, fluorescein staining, cobalt blue light, or manual timing. Measurements of noninvasive breakup time (NIKBUT) obtained with the advanced corneal topographer provide a simple, noninvasive screening test for dry eyes with acceptable sensitivity, specificity, and repeatability [9]. Meibography highlights a glandular architecture, which can be used to analyze glandular density and glandular atrophy [10].

### 2.3. Tear Film Measurements

#### 2.3.1. TMH

TMH was measured twice in each eye using infrared images obtained from the keratograph. The lower tear film meniscus images were captured 5 s after blinking, and the values were graded perpendicular to the lower eyelid margin at the central point.

#### 2.3.2. NIKBUT

NIKBUT was measured twice in each eye using the Oculus noninvasive Keratograph tear breakup time (NIKBUT) tool. The participants were instructed to blink twice before screening and to keep their eyes open to the best of their ability when recording. The NIKBUT was then determined by Keratograph 5M, which automatically generated two measures for NIKBUT: NIKBUT first (NIKBUT-1st) and NIKBUT average (NIKBUT-avg). NIKBUT-1st represents the time point at which the tear film starts to break up. NIKBUT-avg represents the average time for the overall tear film to break up.

### 2.4. Bulbar and Limbal Hyperemia

Increased conjunctival redness is often one of the first signs indicating abnormal strain or pathological changes in the eye. Patients were required to open their eyes as wide as possible and focus on a point inside the camera while a keratograph image was captured. The images were then analyzed by the R-SCAN tool following the evaluation protocol. The software analyzed the image automatically and assigned a red eye index (accurate to 0.1 unit).

### 2.5. Meibography

The upper and lower eyelids were ectropionized, and their respective infrared images were captured. Meibomian gland loss was graded as described by Arita et al. [11]. The meiboscore was as follows: grade 0, no dropout; grade 1, dropout of <1/3 of the lid area; grade 2, dropout of 1/3-2/3 of the lid area; and grade 3, dropout of >2/3 of the lid area. The sum of the upper and lower lid scores was calculated, and the total meiboscore ranged from 0 to 6 [11].

### 2.6. Statistical Analysis

Pearson's chi-squared test was applied to analyze sex and age differences between the groups. The Shapiro–Wilk test or Kruskal–Wallis test was performed accordingly. Normally distributed continuous parameters were analyzed between the groups by one-way ANOVA and Welch ANOVA tests. The Spearman correlation test was used to calculate correlations. All analyses were performed using the statistical software package GraphPad Prism (version 6.00 for Mac). All *P* values less than 0.05 were considered statistically significant. Data are presented as mean ± SD.

## 3. Results

### 3.1. Description of Enrolled Subjects

From April 2016 to January 2017, 120 eyes of 120 type 2 diabetic patients and 40 eyes of 40 nondiabetic patients (22 women and 18 men; aged 64.88 ± 7.04 years) were included in this study. In the diabetic group, the duration of diabetes ranged from 1 to 25 years: in 44 eyes of 44 patients (24 women and 20 men; aged 64.75 ± 8.20 years), the duration was less than 5 years; in 40 eyes of 40 patients (23 women and 17 men; aged 65.03 ± 7.141 years), the duration was 5–10 years; and the duration was more than10 years in 36 eyes of 36 patients (20 women and 16 men; aged 66.11 + 7.44 years).  Table 1 summarizes the demographic and clinical characteristics of the participants. Age and gender did not differ significantly between the subject groups.

### 3.2. Clinical Parameters of the Ocular Surface

**Figure 1:**
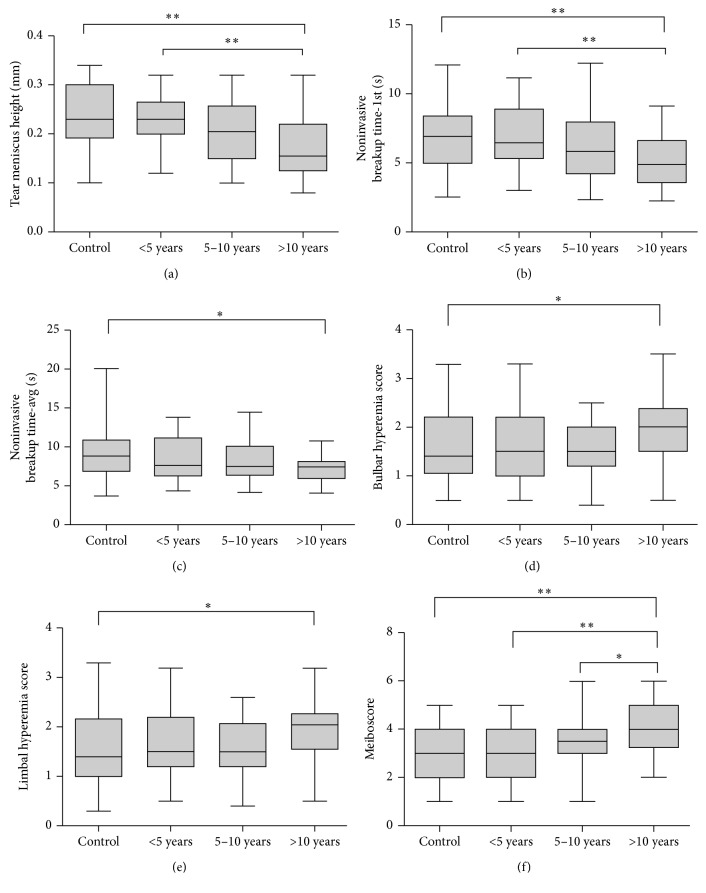
(a) Tear meniscus height (mm) in each group (^*∗*^*P* < 0.05; ^*∗∗*^*P* < 0.01). (b) Noninvasive breakup time first (s) in each group (^*∗*^*P* < 0.05; ^*∗∗*^*P* < 0.01). (c) Noninvasive breakup time average (s) in each group (^*∗*^*P* < 0.05). (d) Bulbar hyperemia score in each group (^*∗*^*P* < 0.05). (e) Limbal hyperemia score in each group (^*∗*^*P* < 0.05). (f) Meiboscore in each group (^*∗*^*P* < 0.05; ^*∗∗*^*P* < 0.01). For all, results are presented as medians and ranges (min to max).

**Figure 2:**
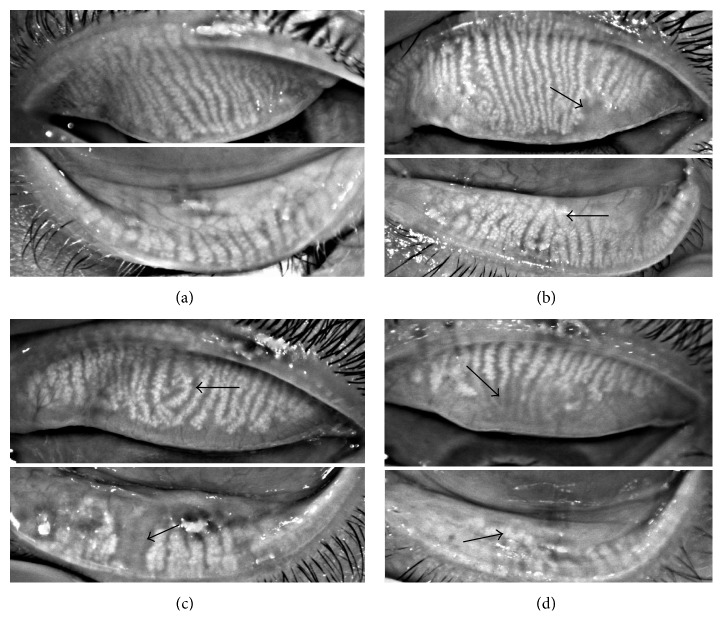
Noninvasive meibographic images of the upper and lower eyelids, respectively. (a) No morphologic changes of meibomian glands in either eyelid were apparent (meiboscore of 0). (b) Minor morphologic changes of meibomian glands in both upper and lower eyelids were apparent (meiboscore of 1). The arrow in the upper eyelid shows the partial absence of meibomian glands, and the arrow in the lower eyelid shows a minor distortion of meibomian glands. (c) Less than 1/3 of the meibomian gland loss and minor morphologic changes in both upper and lower eyelids were apparent (meiboscore of 2). The arrow in the upper eyelid shows partial distortion of meibomian glands, and the arrow in the lower eyelid shows an obvious meibomian gland loss. (d) More than 2/3 of shortening, distortion, and dilation of meibomian glands were observed in both eyelids (meiboscore of 5). The arrow in the upper eyelid shows a large area of meibomian gland loss, and the arrow in the lower eyelid shows a significant distortion and dilatation of meibomian glands.

Medians and ranges of clinical parameters in the control group and diabetic groups are shown in Figures 1(a)–1(f), respectively. Meibomian glands were almost intact in the control group (Figure 2), and only minimal meibomian gland changes were observed in the 5 years and 5–10 years diabetic groups (Figures 2 and 2). In contrast, various meibomian gland changes, including dropouts, shortening, distortion, and dilation were apparent in the over 10 years diabetic patients (Figure 2).

### 3.3. Comparison of Tear Film Parameters and Meiboscore between the Diabetic and Nondiabetic Groups

The results of clinical parameters in each group and *P* values for pairwise comparisons between the groups are presented in Tables 1 and 2. Significant differences were observed in TMH, NIKBUT, bulbar and limbal hyperemia, and meiboscore between the control group and the over 10 years diabetic group.

**Figure 3:**
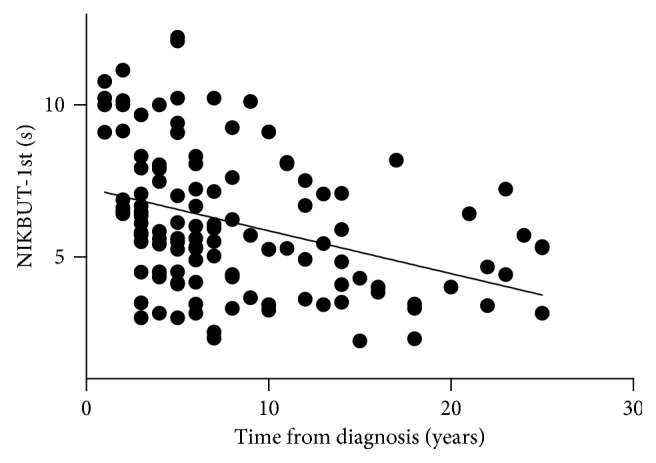
Scatterplot graph showing a slight negative Spearman correlation (*r*_s_=−0.41  and  *P* < 0.0001) between NIKBUT-1st and time from diagnosis.

**Figure 4:**
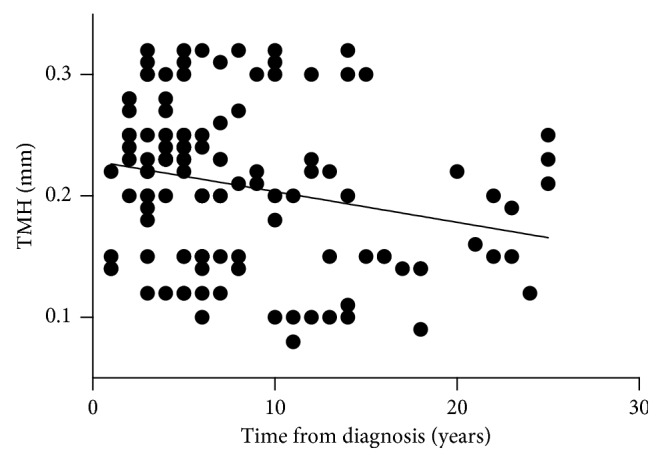
Scatterplot graph showing a slight negative Spearman correlation (*r*_s_=−0.26  and  *P* < 0.01) between TMH and time from diagnosis.

The NIKBUT-1st was significantly shorter in the over 10 years diabetic group compared with the control group (5.13 ± 1.77 versus 6.86 ± 2.20, *P* < 0.01) and the 5 years group (5.13 ± 1.77 versus 6.76 ± 2.24, *P* < 0.01). Similarly, NIKBUT-avg was significantly shorter in the over 10 years diabetic group compared with the control group (7.30 ± 1.63 versus 9.33 ± 3.68, *P* < 0.05). The association between NIKBUT-1st and time from diagnosis was calculated and indicated a slightly negative Spearman correlation (*r*_s_=−0.41  and  *P* < 0.0001; Figure 3). The TMH value was significantly lower in the over 10 years diabetic group compared with the control group (0.18 ± 0.06 versus 0.23 ± 0.06, *P* < 0.01) and the 5 years group (0.18 ± 0.06 versus 0.23 ± 0.05, *P* < 0.01). The association between TMH and time from diagnosis was also calculated and indicated a slightly negative Spearman correlation (*r*_s_=−0.26  and  *P* < 0.01; Figure 4).

The bulbar and limbal redness scan found that more patients in the over 10 years diabetic group showed bulbar and limbal hyperemia compared with the control group (bulbar hyperemia: 1.92 ± 0.66 versus 1.53 ± 0.69, *P* < 0.05; limbal hyperemia: 1.93 ± 0.64 versus 1.53 ± 0.69, *P* < 0.05).

The over 10 years diabetic group showed more meibomian gland changes including dropouts, shortening, distortion, and dilation. The meiboscore in this group was significantly higher compared with the other three groups (the control group versus the over 10 years group, *P*=0.0001; the 5 years group versus the over 10 years group, *P* < 0.0001; and the 5–10 years group versus the over 10 years group, *P* < 0.05), which indicated that the over 10 years diabetic patients suffered a loss of meibomian glands.

In addition to the significant difference in tear film parameters observed between the over 10 years diabetic group and the control group, we also observed a tendency for ocular surface damage in the three diabetic groups as disease duration increased.

### 3.4. Correlations between Clinical Parameters

**Figure 5:**
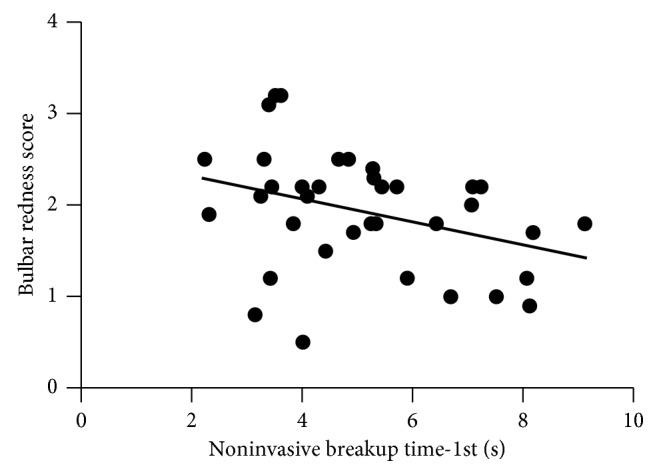
Scatterplot graph showing a negative correlation between bulbar hyperemia and NIKBUT-1st (*r*=−0.36, *r*^2^=0.12, and  *P*=0.039) in the over 10 years group.

In addition to higher bulbar hyperemia and shorter NIKBUT-1st in the over 10 years diabetic group compared with the nondiabetic group, we also found that bulbar hyperemia had a significant negative correlation with NIKBUT-1st in the over 10 years diabetic group (*r*=−0.35  and  *P* < 0.05), which is shown in Figure 5. Thus, the over 10 years diabetic patients who had a less stable tear film were also likely to have higher bulbar hyperemia.

## 4. Discussion

Diabetic eye disease is well known due to its retinal microvascular disorder, diabetic retinopathy. However, diabetes also has an impact on tear film dynamics and can lead to dry eye. The loss of tear film homeostasis in diabetes may be involved and induce dry eye disease [12]. Given that major attention is paid to retinopathy, tear stability changes are merely diagnosed. Therefore, this study was conducted to assess changes of tear film parameters during the course of diabetes and found a shorter tear breakup time, greater conjunctival redness, and severe meibomian gland changes in long-term diabetic patients compared to healthy controls, and a tendency for deterioration was observed with increased disease duration.

Our study found that NIKBUT-1st and NIKBUT-avg in the over 10 years diabetic group decreased significantly compared with the normal control group, reflecting instability of the tear film in these patients. These results correlated with previous studies [13–15]. In addition, the TMH value was significantly lower in the over 10 years diabetic group compared with the control group. A decreasing tendency was also noted in the 5 years diabetic group and 5–10 years diabetic group compared with the control group. Patients with longer duration of diabetes showed reduced TMH and tear film breakup was quicker. Considering that patients often have the disease for a variable amount of time before it is diagnosed, it is really hard to determine the exact disease duration of those patients. We selected data of the two tear film parameters (NIKBUT-1st and TMH) whose *P* value was less than 0.01, and the data of the two parameters were analyzed with time from diagnosis as a linear variable. It showed that the stability of tear film was negatively correlated with the duration of disease in diabetic patients. The result was consistent with the categorical results. Multiple factors in diabetes could contribute to reduced tear film stability. It is possible that damage to the lacrimal gland microvasculature together with autonomic neuropathy may contribute to impaired gland function. In particular, peripheral neuropathy in diabetes leads to abnormalities in corneal nerve density and function [16]. Reduced corneal innervation has been found to correlate with a reduced number of goblet cells and reduced mucin protein [15, 17], resulting in altered tear film.

The bulbar conjunctiva and anterior episclera are nourished by vessels from the anterior and long posterior ciliary arteries [18], and the limbal conjunctiva is supplied by superficial arcades of the anterior ciliary vessels [19]. Our study found that the over 10 years diabetic group showed significant conjunctival redness compared with the healthy controls (*P* < 0.05), and bulbar hyperemia had a significant negative correlation with NIKBUT-1st in the over 10 years diabetic group (*r*=−0.35  and  *P* < 0.05). Conjunctival inflammation is a hallmark of dry eyes [20, 21]. We deduced that increased conjunctival redness may be compensatory to conjunctival inflammation as vasodilation of these vessels results in enhanced blood flow and edema with leakage of fluid and protein from capillaries [22]. In addition, tear evaporation may accelerate when the preconjunctival tear layer temperature rises due to hyperemia. These disorders are associated with signs and symptoms of ocular discomfort such as irritation and foreign body sensation, which can also lead to conjunctival redness.

We compared meibographic findings in the 4 groups, and a significant difference in the meiboscore was found between the control patients and the over 10 years diabetic patients. This was consistent with previous findings [23, 24] where diabetes was associated with MGD. Our data showed that duration of diabetes was closely related to the severity of meibomian gland abnormality (as reflected by the meiboscore, which indicates meibomian gland loss). Several studies have reported that dry eye in diabetes was related to the duration of diabetes [25, 26], but there have been few clinical studies examining meibomian gland function. To the best of our knowledge, the present investigation is the first study to focus on duration of disease in diabetic patients and provides additional evidence for the correlation between the meibomian gland and type 2 diabetes mellitus.

The meibomian gland synthesizes and produces lipids and proteins which form the outermost layer of the tear film. These lipids decrease evaporation and promote stability of the tear film. The International Workshop on Meibomian Gland Dysfunction suggested that MGD is the most prevalent cause of evaporative dry eye and may play a role in aqueous-deficient dry eye [27]. The meiboscore also indicates the thickness of the lipidic layer of the tear film [28], with a higher meiboscore corresponding to a thinner lipidic layer and consequent tear film instability. Although our study provides evidence that meibomian gland function was impaired in long-term diabetic patients, the mechanism of this impairment is unknown and requires further investigation. Recently, a study by Ding et al. demonstrated that insulin stimulated the proliferation of immortalized human meibomian gland epithelial cells (HMGECs), whereas high glucose was found to be toxic to HMGECs [29]. This suggests that insulin resistance/deficiency and hyperglycemia are deleterious to HMGECs, which supports our hypothesis that long-term duration of the disease and insufficient control of blood glucose may be associated with MGD. Moreover, the various medications prescribed to diabetic patients may exacerbate the dry eye state, which may have contributed to the association between duration of diabetes and meibomian gland function in our study. Furthermore, the concomitant inflammatory response in diabetes may also induce MGD. Suzuki et al. suggested that obstructive MGD is a precursor of meibomitis [30].

In addition, Oculus Keratograph 5M is a corneal topographer with additional noninvasive imaging tools for the assessment of tear film kinetics and meibography. It includes the ability to capture clear images of the meibomian gland architecture using infrared light, eliminating the need for instillation of sodium fluorescein into the tear film or the use of white light that exacerbates photophobia in patients with ocular surface disturbance. However, there is no consistent method for meibographic analysis in clinical practice. Our results provide an objective grading method to illustrate complementary data of glandular density.

In summary, our data suggest that long-term type 2 diabetes predisposes to various changes on the ocular surface, which should be noted at an early stage and treated appropriately in order to prevent more severe eye complications. Therefore, close attention should be paid to the ocular surface, especially in long-term diabetics. Further studies are needed to expand the sample size and include fluctuations in blood sugar as a key factor in studying the ocular surface.

## Figures and Tables

**Table 1:** Clinical parameters of the four study groups.

Parameter	Control	<5 years	5–10 years	>10 years
Age (yr)	64.88 ± 7.04	64.75 ± 8.20	65.03 ± 7.14	66.11 ± 7.44
Sex ratio (male/female)	18/22	20/24	17/23	16/20
TMH (mm)	0.23 ± 0.06	0.23 ± 0.05	0.21 ± 0.07	0.18 ± 0.06
NIKBUT-1st (s)	6.86 ± 2.20	6.76 ± 2.24	6.25 ± 2.53	5.13 ± 1.77
NIKBUT-avg (s)	9.33 ± 3.68	8.32 ± 2.63	8.21 ± 2.60	7.30 ± 1.63
Bulbar hyperemia	1.53 ± 0.69	1.57 ± 0.69	1.56 ± 0.57	1.92 ± 0.66
Limbal hyperemia	1.52 ± 0.67	1.57 ± 0.64	1.56 ± 0.55	1.93 ± 0.64
Meibography score	3.15 ± 1.09	3.13 ± 1.08	3.53 ± 1.05	4.25 ± 1.14

**Table 2:** Statistical comparison (*P* values) of clinical parameters among the study groups using the ANOVA test.

Parameter	Control versus <5 years	Control versus 5–10 years	Control versus >10 years	<5 years versus 5–10 years	<5 years versus >10 years	>10 years versus 5–10 years
TMH (mm)	0.9617	0.3517	0.0016	0.6246	0.0061	0.1561
NIKBUT-1st (s)	0.9977	0.6299	0.0056	0.7274	0.0079	0.1327
NIKBUT-avg (s)	0.5321	0.4151	0.0100	0.9958	0.2234	0.3468
Bulbar hyperemia	0.9839	0.9937	0.0490	0.9997	0.0996	0.0912
Limbal hyperemia	0.9726	0.9888	0.0262	0.9995	0.0672	0.0595
Meibography score	>0.9999	0.4259	0.0001	0.3725	<0.0001	0.0241

## Data Availability

The data used to support the findings of this study are available from the corresponding author upon request.

## References

[1] Xu Y., Wang L., He J. (2013). Prevalence and control of diabetes in Chinese adults. *JAMA*.

[2] Ting D. S. W., Cheung G. C. M., Wong T. Y. (2016). Diabetic retinopathy: global prevalence, major risk factors, screening practices and public health challenges: a review. *Clinical & Experimental Ophthalmology*.

[3] DeMill D. L., Hussain M., Pop-Busui R., Shtein R. M. (2016). Ocular surface disease in patients with diabetic peripheral neuropathy. *British Journal of Ophthalmology*.

[4] Bron A. J., De P. C. S., Chauhan S. K. (2017). The ocular surface TFOS DEWS II pathophysiology report. *Ocular Surface*.

[5] Stapleton F., Alves M., Bunya V. Y. (2017). The ocular surface TFOS DEWS II epidemiology report. *Ocular Surface*.

[6] Viso E., Rodriguez-Ares M. T., Abelenda D. (2012). Prevalence of asymptomatic and symptomatic meibomian gland dysfunction in the general population of Spain. *Investigative Ophthalmology and Visual Science*.

[7] Lemp M. A., Crews L. A., Bron A. J. (2012). Distribution of aqueous-deficient and evaporative dry eye in a clinic-based patient cohort: a retrospective study. *Cornea*.

[8] Viso E., Gude F., Rodríguez-Ares M. T. (2011). The association of meibomian gland dysfunction and other common ocular diseases with dry eye: a population-based study in Spain. *Cornea*.

[9] Hong J., Sun X., Wei A. (2013). Assessment of tear film stability in dry eye with a newly developed keratograph. *Cornea*.

[10] Abdelfattah N. S., Dastiridou A., Sadda S. R., Lee O. L. (2013). Non-invasive imaging of tear film dynamics in eyes with ocular surface disease. *Cornea*.

[11] Arita R., Itoh K., Inoue K., Amano S. (2008). Noncontact infrared meibography to document age-related changes of the meibomian glands in a normal population. *Ophthalmology*.

[12] Craig J. P., Nichols K. K., Akpek E. K. (2017). The ocular surface TFOS DEWS II definition and classification report. *Ocular Surface*.

[13] Goebbels M. (2000). Tear secretion and tear film function in insulin dependent diabetics. *British Journal of Ophthalmology*.

[14] Cousen P., Cackett P., Bennett H. (2007). Tear production and corneal sensitivity in diabetes. *Journal of Diabetes and Its Complications*.

[15] Beckman K. A. (2014). Characterization of dry eye disease in diabetic patients versus nondiabetic patients. *Cornea*.

[16] Messmer E. M., Schmid-Tannwald C., Zapp D. (2010). In vivo confocal microscopy of corneal small fiber damage in diabetes mellitus. *Graefe’s Archive for Clinical and Experimental Ophthalmology*.

[17] Dogru M., Katakami C., Inoue M. (2001). Tear function and ocular surface changes in noninsulin-dependent diabetes mellitus. *Ophthalmology*.

[18] Abelson M. B. (2010). Code red: the key feature of hyperemia. *Review of Ophthalmology*.

[19] Van Buskirk E. M. (1989). The anatomy of the limbus. *Eye*.

[20] Stern M. E., Pflugfelder S. C. (2004). Inflammation in dry eye. *Ocular Surface*.

[21] Hessen M., Akpek E. K. (2014). Dry eye: an inflammatory ocular disease. *Journal of Ophthalmic and Vision Research*.

[22] McMonnies C. W. (2017). Conjunctival tear layer temperature, evaporation, hyperosmolarity, inflammation, hyperemia, tissue damage, and symptoms: a review of an amplifying cascade. *Current Eye Research*.

[23] Lin X. L., Xu B., Zheng Y. (2017). Meibomian gland dysfunction in type 2 diabetic patients. *Journal of Ophthalmology*.

[24] Shamsheer R. P., Arunachalam C. (2015). A clinical study of meibomian gland dysfunction in patients with diabetes. *Middle East African Journal of Ophthalmology*.

[25] Manaviat M. R., Rashidi M., Afkhami-Ardekani M., Shoja M. R. (2008). Prevalence of dry eye syndrome and diabetic retinopathy in type 2 diabetic patients. *BMC Ophthalmology*.

[26] Yu L., Chen X., Qin G. (2008). Tear film function in type 2 diabetic patients with retinopathy. *Ophthalmologica*.

[27] Nichols K. K. (2011). The International Workshop on Meibomian Gland Dysfunction: introduction. *Investigative Ophthalmology and Visual Science*.

[28] Eom Y., Lee J. S., Kang S. Y. (2013). Correlation between quantitative measurements of tear film lipid layer thickness and meibomian gland loss in patients with obstructive meibomian gland dysfunction and normal controls. *American Journal of Ophthalmology*.

[29] Ding J., Liu Y., Sullivan D. A. (2015). Effects of insulin and high glucose on human meibomian gland epithelial cells. *Investigative Ophthalmology and Visual Science*.

[30] Suzuki T., Teramukai S., Kinoshita S. (2015). Meibomian glands and ocular surface inflammation. *Ocular Surface*.

